# Lived Experiences of Clinical Nurse Educators Facilitating Interprofessional Simulation: An Interpretative Phenomenological Analysis Study

**DOI:** 10.1111/nicc.70674

**Published:** 2026-09-08

**Authors:** Katherine Hill, Jacqueline McCallum, Chris McAllister

**Affiliations:** ^1^ Nursing & Healthcare School University of Glasgow Glasgow UK; ^2^ Glasgow Caledonian University Glasgow UK

**Keywords:** clinical nurse educator, co‐facilitation, interprofessional simulation education, professional behaviours, qualitative approaches

## Abstract

**Background:**

Interprofessional simulation‐based education is a collaborative approach to learning that enhances communication and teamwork processes that promote safer, more efficient working while providing complex care in a healthcare organisation. While interprofessional simulation‐based education facilitates shared learning among both participants and observers, the extent and nature of this phenomenon as experienced by faculty members remain underexplored.

**Aim:**

To illuminate the lived experiences of clinical nurse educators facilitating interprofessional simulation‐based education to gain deeper insight on how this approach could influence their future practice.

**Study Design:**

A purposive sample of eight clinical nurse educators from Scotland, all with experience working in areas that care for acute and critically ill patients, was recruited to participate in an Interpretative Phenomenological Analysis study. Data were inductively analysed using a systematic, step‐by‐step approach, generating meaningful themes and concepts that can be applied to the context of practice.

**Findings:**

Three master concepts were derived from the interpretative analysis: engagement, synergy and collaboration; the power of the debrief for shared learning; and personal and professional growth. There was a recognition of the significance and importance of working, learning and teaching together.

**Conclusion:**

Facilitating interprofessional simulation‐based education cultivated a shared, new understanding of professional roles and responsibilities, including their respective characteristics, knowledge, skills and behaviours. Specific strategies of co‐facilitation and co‐debriefing create a learning environment that fosters the development of connections and relationships among the faculty and participants, strengthening value and respect in the interprofessional team.

**Relevance to Clinical Practice:**

In critical care, interprofessional simulation‐based education can provide a safe environment for shared learning that strengthens understanding of multidisciplinary roles and promotes effective collaboration in clinical practice. For clinical nurse educators, facilitating interprofessional simulation‐based education aligns with the realities of critical care practices of fostering effective teamwork, enhancing clinical decision‐making and contributing to improved patient safety and outcomes in critically ill populations.

## Impact Statements

- What is known about the topic
  ○ Interprofessional simulation‐based education is a collaborative approach to education that enhances communication, teamwork and clinical decision‐making amongst healthcare professionals within critical care.
  ○ Interprofessional simulation‐based education is shaped by a combination of educational theories that promote active, experiential learning.
- What this paper adds
  ○ The concept of shared learning extends to the facilitators of interprofessional simulation‐based education.
  ○ Facilitating interprofessional simulation‐based education fosters a psychologically safe and collaborative learning environment that enhances interprofessional relationships and role clarity, while strengthening clinical nurse educators' understanding of adult learning theory, ultimately improving both educational and clinical practice. In critical care settings, this approach is particularly valuable given the complexity, acuity and interdependence of care delivery.

## 1 Introduction

Interprofessional simulation‐based education is a collaborative learning approach that strengthens communication, teamwork and clinical decision‐making among healthcare professionals [1]. In critical care, this experiential learning approach provides a safe learning environment in which multidisciplinary teams enhance situational awareness, role clarity and coordinated responses to complex and rapidly evolving patient conditions, thereby optimising care delivery and improving patient outcomes [1]. Published literature highlights the positive experiences of, and impact on, both participants and observers of interprofessional simulation‐based education, effectively bridging the gap between theory and practice [2]. The approach offers a valuable form of experiential learning that strengthens interprofessional collaboration in both undergraduate education and professional practice [3]. However, there is a gap in research exploring how and why shared learning may evolve and influence the clinical and teaching practices of faculty members facilitating interprofessional simulation‐based education.

## 2 Background

Interprofessional learning refers to educational processes in which members of two or more professions learn with, from and about one another [4]. This approach fosters a supportive environment characterised by a shared culture of care, common goals and the integration of professional roles, enabling patient‐centred collaborative practice. Interprofessional working represents the application of these capabilities in practice and does not occur intrinsically; rather, it requires the deliberate development of teamwork, communication and shared decision‐making skills to support effective collaboration in complex clinical settings [5].

In critical care, where safe, high‐quality care depends on effective teamwork and communication, collaborative practice must be actively developed through structured educational approaches [1]. Within this context, simulation‐based education provides an effective platform for interprofessional learning by integrating shared knowledge and skills across professional groups. For example, managing a patient with major trauma requires a well‐coordinated interprofessional approach. While simulation‐based education is inherently multidimensional, its typical structure involves an initial orientation to the session, followed by a simulated clinical scenario and concludes with a post‐simulation analysis through reflective debriefing [6, 7]. Engaging in interprofessional simulation‐based education to rehearse such scenarios enhances teamwork, builds confidence and promotes meaningful interaction between professions, ultimately improving patient care and experience. As such, simulation‐based education is well suited as an educational strategy for interprofessional learning [8].

### 2.1 Educational Theory Informing Simulation‐Based Education

Interprofessional simulation‐based education is shaped by a combination of educational theories that promote active, experiential learning. Kolb's Experiential Learning Theory (1984) [9] supports learning through experience, while Bandura's Social Learning Theory (1977) [10], Tajfel's Social Identity Theory (1979) [11] and Lave & Wengers Learning within Communities of Practice (1991/1998) [12] highlight the importance of social interaction, observation and collaboration. These theories collectively guide interprofessional simulation‐based education, focusing on engagement, reflection and role modelling. Debriefing plays a key role, aligning with a constructivist theoretical framework where the learners individually construct knowledge during the interactive discussion and reflective process [2]. This creates a shared, socially driven learning environment where individuals construct meaning through interaction and collaborative experiences. This learning is not isolated to only the individuals that participate in the simulated scenario but also to the simulation observers who are actively engaged in the debriefing process [2]. It provides the opportunity for the whole group to engage in a reflective discussion, fostering a positive shared learning environment [13]. This reinforces the concept that learning is not a passive but social process, where learners construct knowledge and create meaning from experiences through social activities, engagement and coordination with others [14].

### 2.2 Role of Facilitators in Simulation‐Based Education

Simulation‐based education programmes require a group of facilitators, often referred to as faculty, to be competent in developing scenarios that align with educational learning outcomes and facilitate debrief sessions to enable individuals to reflect on their performance to support new learning that will inform their clinical practice [15]. Simulation‐based education presents an alternative teaching approach that often requires even highly experienced educators to move beyond traditional methods, with the added interprofessional component introducing further complexity. This supports the need for adequate preparation and ongoing development for simulation faculty to adopt this educational methodology as an effective learning tool.

Although there is research that supports the need for faculty development in interprofessional simulation‐based education, there is a dearth of literature on the experiences of the simulation faculty. With simulation‐based education becoming a prominent teaching and learning strategy in the nursing profession, an increasing number of clinical nurse educators (CNE's) are expected to be integral to simulation faculty to support interprofessional learning. The aim of this study was to illuminate the lived experiences of CNE's facilitating interprofessional simulation‐based education to gain deeper insight into how this approach could influence their future clinical and teaching practice.

## 3 Aim

To illuminate the lived experiences of CNEs facilitating interprofessional simulation‐based education to gain deeper insight on how this approach could influence their future practice.

## 4 Methods

### 4.1 Theoretical Framework

Interpretative Phenomenological Analysis (IPA) was chosen as the qualitative research approach for the proposed study as it seeks to illuminate the experiences of CNE's through the interpretation and validation of their unique ‘firsthand’ experiences. Originally rooted in psychology, IPA has become a prominent qualitative research methodology approach which is focused on making sense of personal experiences through the detailed analysis of the phenomenon being studied [17]. Predominantly IPA is an interpretative process that is underpinned by the theoretical axis of phenomenology, hermeneutics and idiography, however, it offers the researcher structured guidance on a systematic in‐depth process of reflective inquiry [18].

### 4.2 Study Setting

The study was conducted in a large National Health Service (NHS) Health Board in Glasgow, Scotland.

### 4.3 Sample

Aligning to IPA methodology, a small, homogenous and purposive sample was recruited [17]. An email was distributed via appropriate gatekeepers, which included a recruitment email and participant information sheet that was forwarded to relevant nursing and simulation education teams to identify interested CNEs. The participants were required to be CNEs with experience facilitating interprofessional simulation‐based education. Snowballing was utilised to identify information‐rich participants to gain access to the specific perspective of the phenomena that was being researched [19]. Participants were CNEs from critical care, emergency medicine, acute medicine, paediatrics and theatre settings. All participants engaged in supporting the care of critically unwell patients, emphasising the relevance of their educational role in the development of safe and effective critical care practice. This adds credibility to the research study as the participants were deliberately chosen on the basis of their experience with the phenomena being investigated [18]. The CNEs interested in participating in this study contacted the researcher, in the first instance, using the information sent via the gatekeeper, which reduced the risk of coercion. Utilising this strategy, the researcher was contacted by and recruited eight CNEs. The sample size of eight aligns to extant IPA research studies, as the number of participants is commonly less than 10 to realistically achieve the detailed interpretative account of individual experiences, which is a fundamental aspect of IPA; this microlevel analysis would not be sufficiently penetrative with a larger sample size [17, 18].

### 4.4 Data Collection

Eight semi‐structured interviews were conducted face‐to‐face in a private meeting room or using Microsoft Teams software between June 2021 and January 2022. Aligning to the interpretative stance of IPA and the importance of researcher immersion, the researcher was the data collection instrument that strengthened the understanding of the lived experiences of the individual [20]. Verbal input from the researcher was minimal [19]; however, strategies such as the use of open‐ended and expansive questions that aligned to the research aim (Table 1), gentle prompting and funnelling were adopted by the researcher to encourage discourse and conversation [17].

**TABLE 1:** Open‐ended interview questions.

Open‐ended interview questions	What are the experiences of CNEs in the facilitation of interprofessional simulation‐based education?
	What are the perceptions of CNEs of shared learning when facilitating interprofessional simulation‐based education?
	How has a collaborative teaching experience contributed to the role of the CNE in their clinical and teaching practices?

### 4.5 Data Analysis

**FIGURE 1:**
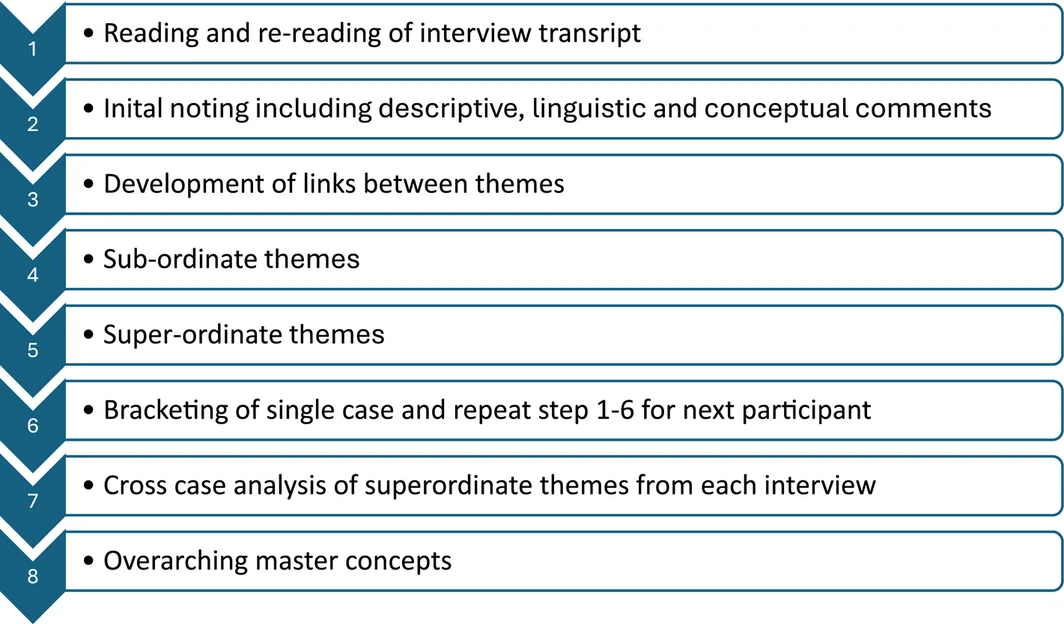
Data analysis process.

The data analysis process in IPA moves from an individual descriptive account to a collective interpretative account, through clustering and thematic analysis, to form a full narrative that accurately represents the phenomena [19]. Interview transcription was undertaken by the researcher as this enhanced researcher immersion and reduced risk of inaccuracies if allocated to a third party [20]. The researcher read a single transcribed interview on multiple occasions, noting any descriptive, linguistic and conceptual observations, capturing and documenting emerging themes that reflected significant aspects of the participants’ account of their experience [18]. The researcher reviewed links and theoretical connections across the emerging themes to form both sub‐ordinate and superordinate themes which required intense interaction between the researcher and transcribed text [17]. The themes from the single case were temporarily ‘bracketed’ and the researcher then progressed on to immersion in the next case, following the same steps [18]. Once each interview was individually analysed, the researcher sought patterns across the superordinate themes to develop overarching master concepts. Figure 1 outlines the data analysis process.

While IPA terminology relating to data analysis was updated by Smith, Flowers and Larkin in 2022, this study remains aligned with the original terminology [19], as the research was initiated before these revisions were published.

### 4.6 Trustworthiness

The Lincoln and Guba [21] evaluation criteria were adopted to assess the trustworthiness of this research study. Credibility checking was adopted through validation of transcribed materials, themes and subsequent research findings by a second expert qualitative researcher, to uphold trustworthiness of the data [22]. A thorough explanation of data interpretation, including thick verbatim descriptions of participant words and meanings, is presented in the research findings which strengthen the transferability of research findings. The outcome of IPA is often considered as ‘giving voice’; therefore, it is fundamental that the participants’ experiences are accurately represented in the data analysis [22]. In addition, conformability was addressed through a meticulous audit trail and a reflective journal [23].

### 4.7 Ethical Considerations

Ethical approval for this study was granted by the university ethics committee on 16 April 2020: HLS/NCH/19/037. The NHS Research and Development department of the health board where the CNEs were being recruited was contacted and it was confirmed that NHS ethics application to the Integrated Research Application System was not required as the researcher was interviewing the CNEs within their professional role. A participant information sheet and consent form were emailed to interested participants to enable them to make an informed judgement to participate. Anonymity of participants was upheld using pseudonyms in both the data storage and the publication of research findings to protect the identity of the participants.

## 5 Findings

### 5.1 Characteristics of Participants

The eight participants were all experienced Registered Nurses with allocated educational responsibility. The length of experience of the participants ranged between 11 and 35 years, with a wide scope of clinical, leadership and educational responsibilities. Participants were CNE's from critical care, emergency medicine, acute medicine, paediatrics and theatre settings, all of whom support the care of critically unwell patients. All participants were trained simulation educators and facilitators of interprofessional simulation‐based education.

### 5.2 Master Concepts

**FIGURE 2:**
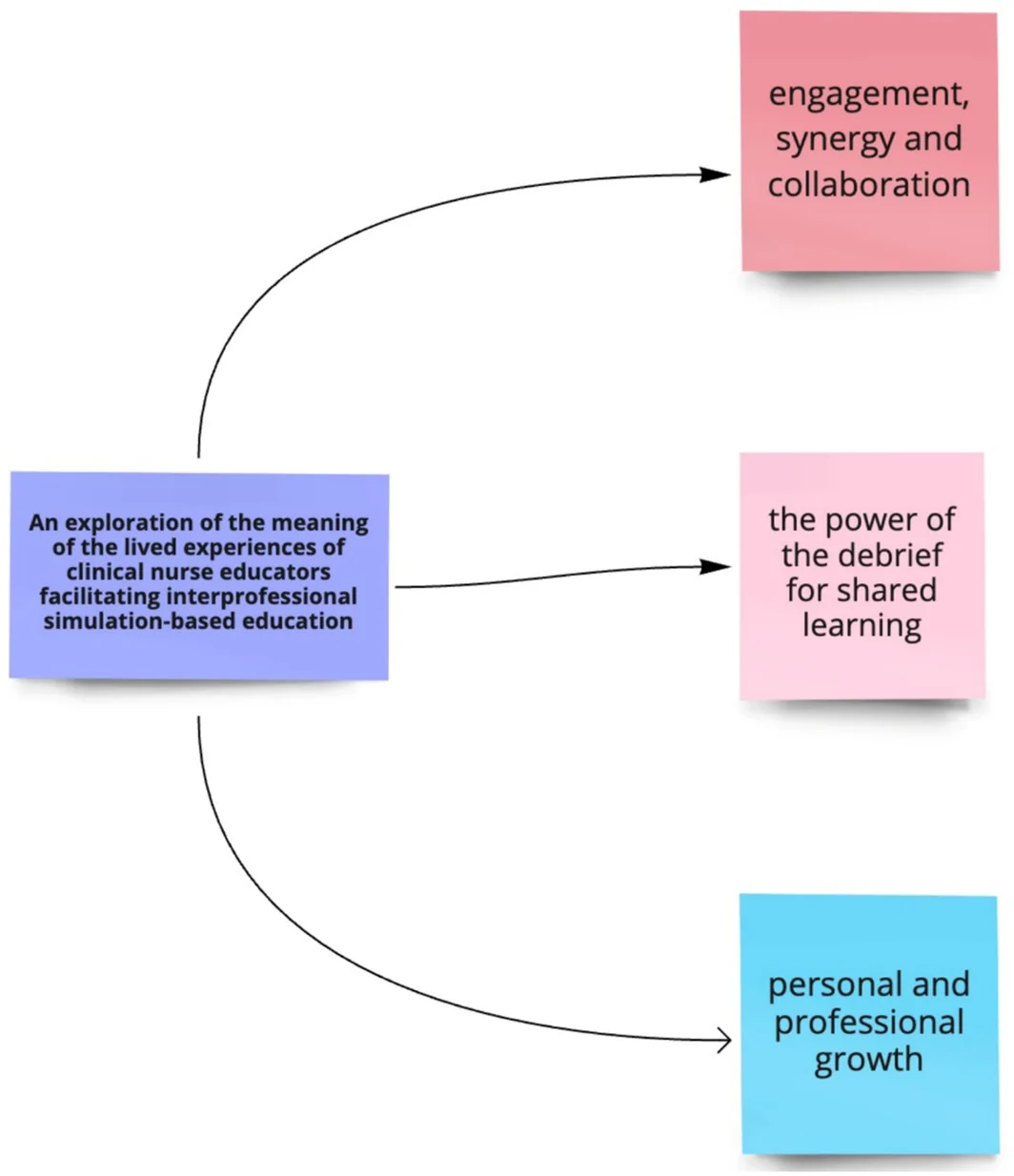
Concept map illustrating three master concepts.

Through the iterative and interpretative process of IPA, three overarching master concepts were developed: engagement, synergy and collaboration; the power of debriefing for shared learning; and personal and professional growth (Figure 2). These concepts represent the researcher's interpretative understanding of participants' accounts while remaining firmly grounded in the lived experiences of the CNEs. Verbatim quotations are included in the narrative to illustrate how the concepts were developed from the participants' narratives, providing transparency and enhancing the credibility and authenticity of the interpretation. Table 2 presents the master concepts and superordinate themes, supported by illustrative participant quotations.

**TABLE 2:** illustrative quotes for master concepts and superordinate themes.

Master concept	Superordinate themes	Participant quotes
Engagement, synergy and collaboration	Learning and support from co‐facilitation Hierarchical culture Value and respect within interprofessional team: ‘looking at things through a different lens’ Work, teach and learn together	*What I believe very strongly is that you need co‐facilitation …they are there to work with you and guide you… you need support* (Participant F) *It encourages people to question… to break down hierarchical barriers [stutter], to think about breaking down hierarchical barriers* (Participant C) *Gaining the experience working alongside other members of the interdisciplinary team in the debrief and the simulation has made me have a greater appreciation of their role* (Participant G) *It allows us to see each other for what we can do, not what our traditional roles are… you have that acceptance and realisation* (Participant E) *There is no point in teaching people in silos… I think when we teach and learn together, we work better together* (Participant E) *100% it is a good thing because we don't work in isolation, so we shouldn't be learning in isolation… we shouldn't be teaching in isolation* (Participant G) *You watch and learn* (Participant D)
The power of the debrief for shared learning	Shared learning through reflection of experiences in debriefing Safe spaces for shared learning through conversation Finding emotional support and psychological safety	*Significance of simulation was actually in the debrief* (Participant G) *Knowledge… knowledge needs to be shared, it absolutely does* (Participant A) *Definitely for us as a team we have, we've learned lots together… so then being able to reflect together has helped us* (Participant C) *And afterwards we'll have like a meta‐debrief…we debrief our debriefs… there is learning in that aswell* (*Participant A*) *We are giving you a safe space to have this discussion* (*Participant E*) *I think it* (*faculty learning*) *happens in that space when you are having a debrief session* (Participant F) *We realised it is not just… educational debrief it's an emotional debrief in a lot of these situation* (Participant E) *Psychological safety is not just about the candidates* (Participant F)
Personal and professional growth	Personal vulnerability, reflection and growth Transferability of knowledge, skills and social behaviours to clinical and educational role Strengthened leadership skills Learning to be an educator	*Facilitation for me has led, led onto coaching* (Participant F) *It is helped me improve my skills in terms of presenting and speaking to wider team* (Participant C) *It's challenging when you first start … you are very conscious… you come from a perspective whereas I'm actually very good at what I do in my real job… so you go back to being a novice… that is quite hard* (Participant D) *It's made me think about the way I am, the way I want to be seen as a practitioner* (Participant C) *I can see the direct relevance to work* (Participant D) *It's improved my individual teaching… I think it has made me a less selfish educator* (Participant E) *It has helped me evolve my teaching style* (Participant G) *I think it's ok to take a step back and take that sort of leader role… delegate within the group… I know it will help them in in the future… I feel I'm in quite good position to step back and actually allow other people to get theses skills and knowledge… and probably that's helped because of simulation* (Participant H) *I talk too much, so I had to stop talking… start listening and start facilitating* (Participant D) *Whereas when I'm working with other professionals who are sim‐based and educator based and have had more experience in medical education that I have, it's good to see styles and get feedback on my debrief and facilitation* (Participant E)

### 5.3 Engagement, Synergy and Collaboration

The CNEs highlighted the value of interprofessional co‐facilitating and co‐debriefing as it enabled them to engage, work synergistically and collaborate with professional colleagues. The CNEs voiced that it was a valuable strategy for shared learning, role modelling and support, thereby strengthening professional respect and effective team‐based behaviours. This collaborative approach not only improves the learning experience for participants, but it also enhances facilitators' own development through the exchange of knowledge and perspectives.

Some CNEs noted that co‐facilitation can help participants feel more represented and engaged, particularly when someone from their own profession is part of the facilitation team:

it's a tribal thing… when there are nurses involved in the debrief the nurses come forward more…it allows us to share our experiences…. (participant E)

Advancing on this a CNE expressed that co‐facilitating with a senior medical colleague offered a sense of security and support during challenging conversations in the debrief:

co‐debriefing…big factor…usually is maybe a nurse and a medic …because you are almost covering both bases…. (participant A)

This reinforces the perception of power hierarchy and perhaps imposter syndrome being present as a CNE faculty member. The influence of hierarchy within healthcare was a recurring theme. However, interprofessional simulation‐based education was recognised as a platform for challenging traditional power dynamics and encouraging more open, collaborative communication. The construct of hierarchy is complex, sometimes acting as a barrier due to associated negative power dynamics that inhibit communication and collaboration, but it also plays a valuable role in realistic clinical education. A few of the CNE's alluded to the presence of nurses in faculty roles as promoting an open dialogue. Simulation sessions, especially debriefs, were described as effective in ‘levelling the playing field’ (participant C) and encouraging all voices to be heard. Despite a growing effort to flatten professional hierarchies in healthcare, a more nuanced view was also offered. A CNE argued that hierarchy can add necessary structure to simulation‐based education scenarios, improving realism during the scenario and reflective debrief: ‘you need hierarchy’ (participant D). Another CNE echoed this, noting that hierarchy supports critical processes like escalation of care, and that simulation facilitation training helps healthcare professionals navigate these structures more effectively through improved communication, thereby enhancing clarity and safe practice.

facilitation development helps you break down barriers…[but it also] aids hierarchy…so, escalation, there is reason hierarchy is there, escalation works and sometimes you need to escalate…. (participant F)

Through facilitating interprofessional simulation‐based education, the CNEs developed a deeper appreciation for the roles and responsibilities of other professions, fostering respect and collaboration. Therefore, rather than eliminating hierarchy, facilitating interprofessional simulation‐based education can improve confidence, empowerment and assertiveness in challenging negative power dynamics in clinical settings.

The benefit of learning from interprofessional colleagues is strongly reflected throughout the interviews, recognised in the opportunities it provides to work, teach and learn together:

…we don't work in isolation, so we shouldn't be learning in isolation…we shouldn't be teaching in isolation…. (participant G)

A CNE recognised that learning is not exclusive to the participants:

…we are here to learn in exactly the same way that our participants are here to learn…. (participant E)

The faculty members are allowed the same permission to learn as the participants of simulation‐based education. The facilitation of interprofessional simulation‐based education had provided them with significant learning opportunities that they embraced.

there's absolutely learning to be had from every scenario; from participants, from faculty…. (participant C)

you learn from…different avenues…different people…. (participant H)

The CNEs reported that working closely with different professions during simulation‐based education enhanced their understanding of others' contributions, which was transferable to clinical practice. They described a shift from siloed thinking to more blended, team‐based approach to education and clinical practice. Healthcare is inherently team‐based, and simulation‐based education helps build trust, encourage open dialogue and enhance team dynamics.

### 5.4 The Power of Debrief for Shared Learning

The debrief was viewed as an educational and social learning ‘space’ where the participants and faculty feel physically, emotionally and psychologically safe. It is a psychologically ‘safe container’ that promotes open and honest collaborative discussions, often illuminating interprofessional vulnerabilities which can contribute to breaking down the barriers associated with traditional hierarchal power structures. Faculty development activities such as the ‘meta‐debrief’ were an opportunity to reflect on ‘where you are as a facilitator’ (participant F). It was a space that empowered them, as learners, to engage in a reflexive discussion, peer feedback and evaluation of their practice that supported the development of their facilitation skills. This process is founded on a shared, inclusive, honest and constructive discussion that facilitates professional development and contributes to the building of positive connections and relationships within the interprofessional team.

it's almost like “this is a safe space, we are here to educate, if we make mistakes, we are here to learn from them” … it's almost giving you the same license you give to your participants that we are here to learn…. (participant E)

It was repeatedly noted that the CNEs acknowledge the impact and influence of learning from other faculty members' debriefing experience, skills and behaviours, with one CNE describing the process as gaining ‘little nuggets’ (participant D) of insight.

I've learned from my medical colleagues …how they teach, how they do the debrief…. (participant G)

Learning is not linear, rather an evolving process, that is influenced by multiple factors such as observation, experience and critical reflection.

### 5.5 Personal and Professional Development

Undertaking the role as a facilitator of interprofessional simulation‐based education has made a positive contribution to the CNEs' role in their educational and clinical practices, contributing to personal growth and development.

the one win from developing facilitation skills is they are so transferable … they sit in every single element of healthcare…. (participant F)

The CNEs regarded the participation in educational opportunities such as faculty development courses, peer feedback and professional development activities as fundamental to enhance the transition and adaption to their role as an educator. Faculty development courses and the observation of more experienced simulation educators enabled the CNEs to respect the importance of developing an understanding of educational theory and adult learning strategies to facilitate a more learner centred pedagogical approach to their wider teaching practices.

we're actually learning all the time… it's about being an educator…. (participant F)

This learning process enabled them to develop their professional identity as an educator, which is different to their professional identity as a clinical nurse. The development of knowledge, skills and experience as an educator in facilitating interprofessional simulation‐based education has positively influenced their clinical practice. The CNEs had increased their confidence in their own leadership skills, with a recognition of the importance of non‐technical skills such as communication and assertiveness in collaborative practice.

facilitating simulating has only really led to more positive communication within the IP team…. (participant H)

Overall, the experiences of the CNEs facilitating interprofessional simulation‐based education empowered them with the knowledge, skills and values to transform their clinical and educational practices. In addition, it has increased their confidence and motivation in their commitment to other professional development.

## 6 Discussion

Interprofessional simulation‐based education is widely recognised as an effective experiential learning approach that enhances collaborative practice among healthcare professionals [1, 3, 24, 25, 26, 27]. In critical care settings, this is of particular importance due to the requirement for a rapid and coordinated response to patient deterioration and management, whereby effective interprofessional collaboration is fundamental to optimising patient outcomes [1]. This study contributes new insights by exploring CNEs' experiences as facilitators in interprofessional simulation‐based education, emphasising the importance of respect, professional identity, hierarchical culture and co‐facilitation, all of which underpin effective interprofessional collaboration within complex critical care environments. Findings indicate that facilitating interprofessional simulation‐based education promotes mutual respect, understanding and patient‐centred care by fostering shared learning and role clarity within a safe environment. CNEs described the experience as transformative, improving perceptions of professional roles and strengthening team‐based behaviours. Therefore, this safe learning space allows facilitators to strengthen their clinical decision‐making skills, communication strategies and team dynamics without risk to patients. Through facilitating interprofessional simulation‐based education, there is a clearer understanding of the roles, responsibilities and expertise of other professionals within the multidisciplinary team, which strengthens interprofessional relationships and promotes more effective collaboration in the clinical environment, which ultimately contributes to improved patient safety and quality of care.

The study highlights that while strong uniprofessional identities provide belonging, they can hinder collaboration. Interprofessional simulation‐based education supports identity formation within an interprofessional context, encouraging active knowledge sharing and social learning [10, 12]. This approach challenges professional tribalism and hierarchical norms which are well recognised in the critical care environment as contributing to power imbalances, communication barriers and stereotypes that can impede teamwork [16, 28]. Although hierarchy is often viewed negatively, the CNEs in this study perceived it as necessary for maintaining patient safety, particularly during time‐critical clinical emergencies where clear leadership and rapid decision‐making are required. Within critical care practice, hierarchy can therefore serve an important functional role when it supports coordination and accountability. Importantly, the development of facilitation skills, such as improving understanding and communication between professions, enables collaboration that works with rather than against hierarchical structures. This aligns with evidence from critical care simulation literature demonstrating that structured interprofessional training enhances communication, leadership and team‐based behaviours in high‐acuity environments [1]. Consequently, when hierarchy is constructively integrated into interprofessional education and practice, it can contribute to a respectful working environment characterised by psychological safety, where all professional voices are valued and heard, ultimately strengthening team performance and patient care in critical care settings.

Co‐facilitation enables facilitators to model respectful teamwork and shared decision‐making while integrating diverse professional expertise and perspectives [13, 29]. Through a social identity lens, it disrupts rigid group boundaries and fosters a shared team identity. This is fundamental in high‐risk critical care environments, where rapid decisions and interdependence demand psychological safety [30]. Hierarchical barriers can inhibit speaking up and delay escalation, risking patient harm [31]. This study substantiates that interprofessional simulation‐based education can foster inclusive identities, encourage open communication and cultivate mutual respect, thereby supporting interprofessional socialisation. This strengthens team behaviours and relationships, ultimately contributing to safer, more effective patient care.

This study's findings further attest to simulation‐based education as an innovative teaching strategy that challenges the most experienced educators [16], with the interprofessional aspect adding a layer of complexity [32]. Effective facilitation involves managing multiple roles and navigating interprofessional dynamics, emphasising the need for ongoing faculty development to build confidence, knowledge and skills [33]. The power of simulation as a learning tool is dependent on the understanding and application of underpinning pedagogical educational practices, identifying the need for the facilitators of interprofessional simulation‐based education to understand foundational principles of this innovative teaching strategy such as social constructivism, experiential learning and social learning theory [34]. This highlights the depth and complexity of learning that is required for faculty development, and the transition from experienced clinician to educator. They learned through observation, social interactions and peer critique, aligning with the concept of vicarious learning [34, 35]. The CNEs expressed that they were ‘learning to be an educator’, acknowledging the importance of developing an understanding of educational theory and adult learning strategies to facilitate a more learner centred pedagogical approach to the facilitation of interprofessional simulation‐based education and their wider teaching practices. From a critical care nursing perspective, this is important as understanding underpinning educational theory supports the development of learner‐centred approaches that enhance clinical reasoning, decision‐making and teamwork. Strategies such as reflective debriefing, when embedded following clinical events, facilitate active engagement, enhance communication and promote psychological safety. This, in turn, supports experiential learning and the effective transfer of knowledge into practice, ultimately contributing to improved patient safety.

This developmental process is non‐linear, influenced by cognitive, sociocultural and organisational factors [36]. From the perspective of an educator facilitating simulation‐based education, Benner's Novice to Expert model, adapted by Cheng et al. [37] (Table 3), supports the developmental journey of educators in acquiring debriefing skills over time. The CNEs in the study acknowledged that they started as novice facilitators, despite extensive clinical experience, and emphasised how experiential learning, reflective practice and critical thinking were central to their growth.

**TABLE 3:** Conceptual framework for debriefing (Cheng et al. 2020).

Benner's stages of clinical competence	Cheng et al. (2020) conceptual framework for debriefing
Novice	Discovery stage
Advanced beginner	
Competent	Growth stage
Proficient	Maturity stage
Expert	

Aligning to the key‐shared concepts of adult learning theory, the CNEs are engaging in the learning process through experience and reflexivity to develop their skills in facilitation to create a meaningful space for collaborative learning [38]. This can be transformative for their teaching practice, enabling them to adopt more learner‐centred approaches, promote reflective discussions and support the development of critical thinking skills that are essential in critical care settings. This learning process supported the development of their professional identity as educators, distinct from their identity as clinical nurses; however, these identities are not mutually exclusive and can be integrated within their role.

Critical care delivery is characterised by complexity, urgency and interdependence between interprofessional groups. Facilitation of interprofessional simulation‐based education contributed to the development of the CNEs' pedagogical and experiential expertise, enhancing their confidence in leadership and highlighting the significance of non‐technical skills, such as communication and assertiveness, within interprofessional practice. These experiences were perceived as transformative, equipping educators with the knowledge, skills and professional values required to inform and advance both their clinical and educational practice.

### 6.1 Limitations

This research study interviewed a small, purposive sample of eight CNEs which is regarded as appropriate and important for IPA methodology to obtain rich, textured and meaningful insight into the phenomenon being investigated, allowing the reader to determine the theoretical transferability of findings to their own practice. It is important to note that participant demographics vary, as the CNEs have different lengths of experience both as Registered Nurses and as simulation‐based education facilitators, which may have led to differences in their perspectives. Despite variability within the sample, participants were all experienced simulation‐based educators that had clinical experience working within interprofessional teams caring for acute and critically unwell patients. The study is strengthened with the thick and detailed interpretation of data focusing on participants' words and meanings, enhancing the credibility and transferability of the findings [19].

### 6.2 Recommendations for Further Research

It would be advantageous to undertake further research exploring the experiences of other disciplines that form the interprofessional faculty facilitating interprofessional simulation‐based education. This will enable the comparison of findings between different professions, providing further insight and perspective into the dynamics of learning within interprofessional practice, thereby strengthening transferability of findings to wider practice.

### 6.3 Implications for Policy and Practice

It has been identified in this study that simulation‐based education should be facilitated by an interprofessional faculty to create a supportive learning environment that reduces the risk of intimidation, negating the power dynamics associated with hierarchy which can impede educational opportunities among both the participants and the faculty. Adherence to a national standardised faculty development framework and attendance at continuing professional development workshops should become an integral and mandatory aspect of the personal development plan and review (PDPR) of CNEs facilitating simulation‐based education. From an organisational perspective, this will contribute to a standardised approach to the development of CNEs in facilitating simulation‐based education. Competent and appropriately trained simulation faculty will contribute to the enhancement of team performance, facilitating the effective translation of learning into clinical practice within critical care settings.

## 7 Conclusion

Interprofessional simulation‐based education creates a psychologically safe environment that facilitates shared learning among facilitators, strengthening interprofessional relationships and collaborative practices, which is particularly significant within critical care where effective teamwork underpins safe, high‐quality patient care. Facilitating interprofessional simulation‐based education fosters a shared understanding of professional roles and responsibilities, encompassing associated knowledge, skills and behaviours, alongside a recognised value of collaborative working, learning and teaching. Furthermore, the CNEs expressed that their experiences had enabled them to gain a deeper, theory‐informed understanding of adult learning, which has been transformative in shaping both their educational and clinical practice.

## Author Contributions

**Katherine Hill:** conceptualisation, methodology, validation, formal analysis, investigation, data curation, writing – reviewing and editing, writing – original draft, visualisation, project administration. **Jacqueline McCallum and Chris McAllister:** validation, formal analysis, data curation, writing – reviewing and editing and supervision.

## Funding

This study received funding from NHS Greater Glasgow & Clyde (NHS GGC) staff bursary and The General Nursing Council for Scotland (Education) Fund (1983) for contribution to Professional Doctorate university fees.

## Ethics Statement

Ethical approval for this study was granted by the university ethics committee: HLS/NCH/19/037. The NHS Research and Development department of the health board where the CNEs were being recruited was contacted and it was confirmed that NHS ethics application to the Integrated Research Application System was not required as the researcher was interviewing the CNEs within their professional role. A participant information sheet and consent form were emailed to interested participants to enable them to make an informed judgement to participate. Anonymity of participants was upheld using pseudonyms in both the data storage and the publication of research findings to protect the identity of the participants.

## Conflicts of Interest

The authors declare no conflicts of interest.

## Supporting information


**Data S1:** Checklist.

## Data Availability

The data that support the findings of this study are available on request from the corresponding author. The data are not publicly available due to privacy or ethical restrictions.
